# The ICCAM platform study: An experimental medicine platform for evaluating new drugs for relapse prevention in addiction. Part B: fMRI description

**DOI:** 10.1177/0269881116668592

**Published:** 2016-10-04

**Authors:** John McGonigle, Anna Murphy, Louise M Paterson, Laurence J Reed, Liam Nestor, Jonathan Nash, Rebecca Elliott, Karen D Ersche, Remy SA Flechais, Rexford Newbould, Csaba Orban, Dana G Smith, Eleanor M Taylor, Adam D Waldman, Trevor W Robbins, JF William Deakin, David J Nutt, Anne R Lingford-Hughes, John Suckling, ICCAM Platform

**Affiliations:** 1 Centre for Neuropsychopharmacology, Division of Brain Sciences, Imperial College London, London, UK; 2 Neuroscience and Psychiatry Unit, Institute of Brain, Behaviour and Mental Health, The University of Manchester, Manchester, UK; 3 Department of Psychiatry, University of Cambridge, Cambridge, UK; 4 Behavioural and Clinical Neuroscience Institute, University of Cambridge, Cambridge, UK; 5 Imanova Limited, London, UK; 6 Centre for Neuroinflammation and Neurodegeneration, Division of Brain Sciences, Imperial College London, London, UK; 7 Department of Psychology, University of Cambridge, Cambridge, UK; 8 Cambridgeshire and Peterborough NHS Foundation Trust, Fulbourn, UK

**Keywords:** Brain, human, magnetic resonance imaging, substance-related disorders

## Abstract

**Objectives:**

We aimed to set up a robust multi-centre clinical fMRI and neuropsychological platform to investigate the neuropharmacology of brain processes relevant to addiction – reward, impulsivity and emotional reactivity. Here we provide an overview of the fMRI battery, carried out across three centres, characterizing neuronal response to the tasks, along with exploring inter-centre differences in healthy participants.

**Experimental design:**

Three fMRI tasks were used: monetary incentive delay to probe reward sensitivity, go/no-go to probe impulsivity and an evocative images task to probe emotional reactivity. A coordinate-based activation likelihood estimation (ALE) meta-analysis was carried out for the reward and impulsivity tasks to help establish region of interest (ROI) placement. A group of healthy participants was recruited from across three centres (total *n*=43) to investigate inter-centre differences.

**Principle observations:**

The pattern of response observed for each of the three tasks was consistent with previous studies using similar paradigms. At the whole brain level, significant differences were not observed between centres for any task.

**Conclusions:**

In developing this platform we successfully integrated neuroimaging data from three centres, adapted validated tasks and applied whole brain and ROI approaches to explore and demonstrate their consistency across centres.

## Introduction

Addiction is a major global health problem, with illicit drug and alcohol use disorders contributing to approximately 20% of the burden from mental health disorders (Whiteford et al., 2013). Of concern is the lack of effective interventions for these disorders, whilst the prevalence of alcohol, opioid and cocaine addiction is increasing (Lingford-Hughes et al., 2012; Whiteford et al., 2013). The growing knowledge about the brain mechanisms underpinning addiction offers an important opportunity to develop new treatments. Studying the neurobiology of addiction can be challenging due to its common relapsing–remitting clinical course. To address this, a collaboration between Imperial College London, the University of Cambridge and the University of Manchester (ICCAM; http://www.bbmh.manchester.ac.uk/ICCAM/) was formed under a Medical Research Council (MRC) addiction initiative to maximize the existing magnetic resonance imaging (MRI) and clinical infrastructure and expertise available in the UK. Establishment of a platform is necessary to provide suffi-cient throughput to rapidly evaluate potential pharmacological treatments in addiction to allow us the best chance of meeting this area of significant unmet need. Here, the term ‘platform’ refers to the concept of applying a framework of experimentation (i.e. the functional MRI (fMRI) tasks and associated measures) and analysis that can be applied under different conditions and on different groups to accelerate efforts to identify effective treatments for challenging diseases (Berry et al., 2015).

The rationale for the cognitive processes and neuropharmacology as well as the clinical population studied in the ICCAM platform have been described in detail elsewhere (Paterson et al., 2015). Briefly, the aim was to develop a neuroimaging platform to assess candidate brain pathways underpinning addiction and relapse using appropriate fMRI tasks, and assessing their modulation by different pharmacological challenges (antagonists of Dopamine Receptor D3 (DRD3), µ-opioid receptors and Neurokinin 1 (NK1) receptors) in alcohol, heroin and cocaine addiction. Here we describe the establishment of fMRI tasks in the three centres in healthy volunteers and investigate their properties. Results from the ICCAM platform with regard to brain responses to the tasks in addicts and modulation to pharmacological challenges will be reported elsewhere.

In addiction, common themes implicated in relapse involve difficulties with reward or motivation and impulse control, as well as stress-related emotional reactivity. There is a considerable body of evidence from neuroimaging studies that a dysregulated reward/motivation system in addiction as well as deficits in inhibitory control, poor decision making (Loree et al., 2014; Noel et al., 2013) and stress (Koob and Kreek, 2007; Sinha and Li, 2007) contribute to relapse. We therefore selected established fMRI tasks designed to elucidate the neural responses associated with these processes – reward/motivation, impulse control and emotional reactivity.

For reward, we chose the widely used monetary incentive delay task since it provides a measure of reward sensitivity with robust increases in striatal activity evident in healthy volunteers (Knutson et al., 2001). Striatal activity has been shown to be reduced in alcohol dependence (Wrase et al., 2007b), and in stimulant use related to treatment status (Bustamante et al., 2014; Jia et al., 2011; Schouw et al., 2013). Furthermore, ventral striatal activation in response to the task is sensitive to pharmacological modulation by amphetamines (Knutson et al., 2004), olanzapine (Schlagenhauf et al., 2008) and catecholamine depletion (Hasler et al., 2009).

For impulsivity, we chose the go/no-go task since it provides a measure of inhibitory control mediated by prefrontal–striatal circuits (Garavan et al., 2002, 2003). Neural responses during go/no-go have been shown to be altered in cocaine users (Connolly et al., 2012; Kaufman et al., 2003) and opiate addiction (Forman et al., 2004), and to be modulated by certain dopaminergic gene variants in heavy drinkers (Filbey et al., 2012).

To explore stress we exploited the associated emotional dysregulation, since amygdalar response is robustly observed and altered in a range of neuroimaging studies of addicts (Asensio et al., 2010; Gilman and Hommer, 2008; Li and Sinha, 2008). Therefore, in common with others, we used an evocative images task to assess emotional reactivity to contrasting aversive images with neutral images from the International Affective Picture System (IAPS) library. Photographs containing scenes of animate and inanimate objects or scenes were displayed in a block design, with each block containing either neutral or distressing images of an injurious or threatening nature. In addition, due to studying addiction to different substances and therefore potentially variable cue-reactivity, images had no explicit alcohol/drug content. Due to time constraints within the imaging session, positive images were not included. Similar tasks have been shown to elicit amygdala responses and have been employed to demonstrate enhanced responses in alcohol dependence (Gilman and Hommer, 2008) that were decreased by an NK1 receptor antagonist (George et al., 2008). Whilst salient cues are strong triggers for relapse, we did not include such a task due to concerns about determining and optimizing salience for each participant, ensuring salience was equal across different substances, and habituation over five sessions. We also had to consider the time constraints of our imaging sessions which were developed to be tolerable for participants such that they would perform adequately.

In order to define a priori where responses were expected in the brain for each task, we carried out a coordinate-based meta-analysis of neuroimaging data using activation likelihood estimation (ALE) (Eickhoff et al., 2009, 2012; Turkeltaub et al., 2002, 2012). Such an approach overcomes potential bias of choosing regions of interest (ROIs) based on an investigator’s knowledge of where a task has been found to modulate activity in their previous work. For instance, several studies have used these methods to establish locations of consistent response to reward (Bartra et al., 2013; Keuken et al., 2014), impulsivity (Criaud and Boulinguez, 2013; Simmonds et al., 2008) and emotional reactivity (Fusar-Poli et al., 2009). However, there is much variation in the specifics of fMRI tasks, even amongst those considered as ‘standard’, with meta-analyses often using relatively broad inclusion criteria. These have the advantage of increased statistical power at the expense of reduced specificity. Here we seek to establish not only the general neural correlates of the paradigms under investigation, but also those elicited by the specific versions of each task as they were implemented.

Whilst the advantages of multi-centre study designs are numerous and well-rehearsed (Paterson et al., 2015), the involvement of multiple acquisition centres introduces new factors that require appropriate consideration during subsequent analysis. In particular, the overall variance is inflated by a between-centre factor, and there is potential for bias should a sub-set of centres have significantly greater statistical power than the others.

In this paper, therefore, we detail both the specific versions of the tasks used in the platform fMRI study, along with their modelling, sufficiently to enable replication. Following this, and taking each task in turn, we establish their characteristics before investigating inter-centre differences.

## Methods

The study was conducted in accordance with the Declaration of Helsinki. Ethical approval was obtained from West London and Gene Therapy Advisory Committee (GTAC) National Research Ethics Service (NRES) committee and relevant Research Governance and Participant Identification Centre approvals obtained. Data were collected at three UK centres: Imanova Limited, London; The Wolfson Brain Imaging Centre, University of Cambridge; and Salford Royal NHS Foundation Trust, Manchester.

### Participants

Out of the 155 participants who had a full baseline imaging session in the main ICCAM study, 68 were healthy controls with no history of drug or alcohol dependence (19, 33 and 16 from London, Cambridge and Manchester, respectively) – only this group is examined further here. These were recruited from healthy volunteer databases, via multimedia advertising including fliers, posters, social media, local newspapers, websites, homepages and via word of mouth.

From this group of 68 a subgroup of 43 (*n*=15, 15 and 13 from London, Cambridge and Manchester, respectively) were chosen so that each centre had a similar distribution of gender and age. This group of 43 healthy individuals was used for both the task characterization and inter-centre variability investigations. Although not reported in this work, the majority of these participants took part in further imaging sessions in the main ICCAM study, beyond these baseline sessions.

### fMRI task protocols

E-Prime 2.0 RC (version 2.0.8.90) was used to run all tasks. Tasks were adapted such that two runs of each (along with resting state and preliminaries) could be achieved within a one hour period.

### Monetary incentive delay task

The monetary incentive delay task, designed to probe reward sensitivity, was modified from Knutson et al. (2001). Participants could win or lose money depending upon how quickly they reacted to a target stimulus. The task contained win, lose and neutral trials. For the win trials, participants could win £0.50 if they responded quickly enough; for the lose trials, participants lost £0.50 if they did not respond quickly enough; and for the neutral trials participants neither won nor lost money. During each run, 216 volumes were collected, for a run length of 7 min 12 s.

This task used an event related design, though with long mini-blocks (i.e. several TRs (repetition time) in length), carried out in two runs (for a combined length of 14 min 24 s). Each run contained 18 win trials, 18 neutral trials, and six lose trials. In total, the task contained 36 win trials, 36 neutral trials and 12 lose trials. The task was set to obtain approximately 66% accuracy for the win trials. Furthermore, the task was designed to give an approximate winnings total of £10 (a perfect, though unlikely, result would result in winnings of £18).

Participants were informed as to what trial they were about to perform via ‘cues’ that appeared on the screen for one second. Following the cues, there was an anticipation period (i.e. a blank black screen) before the target stimulus was presented. The duration of the anticipation period was randomly selected as 2, 3 or 4 s (with equal numbers of each period for each trial type). The anticipation period was immediately followed by the presentation of the target stimulus. The duration of the target stimulus differed depending upon the accuracy of participants.

The starting duration for the win and neutral trials was 280 milliseconds (ms) for both runs (i.e. the time allowed for a participant to press the button after the stimulus was displayed). For the individualized algorithm, if a participant responded in time for the target stimulus, the target duration dropped by 10 ms (until the floor duration of 150 ms was reached). If a participant missed a trial, the target duration increased by 10 ms (until the ceiling duration of 300 ms was reached). The duration of each of the target symbols for each trial (win, neutral, lose) was contingent upon the participant’s accuracy for the same trial type only – that is, win trial accuracy only affected stimulus duration of subsequent win trials, and not neutral or loss trials. Participants were informed if they were successful immediately (always 0.5 s after target presentation) after each trial, together with a display of their total winnings, which was shown for 2 s. For each trial type the interval between the end of this information/winnings display, and the onset of the next cue were 2.4, 3.4, or 4.4 s, with equal numbers of each period across trial types.

The starting duration for the loss trials was 240 ms for both runs. A reduced loss starting duration was chosen as we required participants to lose in order to increase the incentive salience of reward trials. A fixation cross was displayed for 12 s at the beginning of each run.

### Go/no-go task

The go/no-go task, designed to probe impulsivity, was modified from Garavan et al. (2002). Participants were presented with an alternating series of letter Xs and letter Ys and asked to ‘respond as quickly as possible’ to the appearance of each letter presented (‘go’ trial), except when the alternating sequence was broken by the appearance of a letter the same as that presented previously (‘no-go’ trial). During each run, 131 volumes were collected, for a run length of 4 min 22 s.

This task used an event related design and was carried out in two runs. Each run contained 250 trials. 220 of these were ‘go’ trials where participants had to respond, and 30 of these trials were ‘no-go’ trials where the participant had to withhold a response (i.e. when the letter was the same as the previous letter). On average there was one ‘no-go’ trial every 8 s (range: 4–14 s).

Each letter was presented on the screen for 900 ms and was followed by a 100 ms inter-stimulus interval consisting of a blank screen. A fixation cross was displayed for 12 s at the beginning of each run.

### Evocative images task

The evocative images task was designed to probe emotional reactivity. Participants were presented with aversive IAPS images containing scenes of injury or threat and neutral IAPS images containing scenes of animate and inanimate objects. Participants had to press their response pad to each image to ensure they were awake and attending to the images. During each run, 196 volumes were collected, for a run length of 6 min 32 s.

The task used a block design and was carried out in two runs. Each run contained four blocks of aversive images and four blocks of neutral images. Each block contained six images and each block was separated by a rest period to prevent carry-over effects. Images in each block were presented in a pseudorandomized order. The second run of the task contained the same images as the first run, but presented in a different order. Due to possible habituation effects, different images were presented at each session.

Each block was 32.4 s (six images of 5 s duration followed by a 400 ms inter-stimulus interval). Each rest period lasted 15 s. A fixation cross was displayed for 12 s at the beginning of each run.

### Activation likelihood estimation meta-analyses

To identify appropriate regions of interest (ROIs) for specific analyses, activation likelihood estimation (ALE) meta-analyses (Eickhoff et al., 2009) of the literature were carried out using the BrainMap Project’s GingerALE version (2.3.1) for both the monetary incentive delay and go/no-go tasks.

The following general study selection criteria were applied: (1) participants’ mean age greater than 25 years (to preclude those studies focusing on young adults or children); (2) used only one form of response (i.e. a single button for input); (3) reported activation foci in either Talairach or Montreal Neurological Institute (MNI) space; (4) published in English; (5) appeared in a peer reviewed publication; (6) used human participants; (7) used greater than six participants; (8) published between January 2002 and April 2013. Only healthy control data was used if a study included other groups.

Monetary incentive delay studies were identified by searching the PubMed database using the terms: (‘monetary’ OR ‘money’ OR ‘anticipation’) AND (‘fMRI’ OR ‘neuroimaging’); and by searching the BrainMap database (Fox et al., 2005) using the filters ‘fMRI’ and ‘reward’. In order to identify previous studies with comparable versions of the monetary incentive delay task examined here, these further criteria were used: (1) participants actually paid their winnings; (2) loss trials present; (2) more than ten gain trials; (4) reward anticipation modelled against neutral anticipation.

Go/no-go studies were identified by searching the PubMed database using the terms: (‘go/no-go’ OR ‘response inhibition’) AND (‘fMRI’ or ‘neuroimaging’); and by searching the BrainMap database using the filters ‘fMRI’ and ‘go/no-go’. In order to identify previous studies with comparable versions of the go/no-go task examined here, these further criteria were used: (1) no-go trials make up fewer than 40% of all trials; (2) not include an oddball stimuli (i.e. not include go trials with a different letter/shape/image); (3) use only letters (not images); (4) use only one no-go cue; (5) correct no-go modelled against either correct go or an implicit baseline.

For studies that reanalysed previously used data, only the original studies were used. All coordinates were transformed into MNI space as necessary. ALE was performed for each task with a False discovery rate (FDR) of *p*<0.05 (corrected) and a minimum cluster volume of 600 mm^3^ (0.6 ml).

For each task, an ROI was made up of two 5 mm radius spheres placed bilaterally such that they overlapped with the weighted centre coordinates of the strongest bilateral ALE clusters, while robustly covering grey matter.

### Defining regions of interest: evocative images task

Although emotional imaging tasks have been used in many previous studies, the considerably variability in the design (especially in the specific images used within) precludes a meta-analysis using the criteria used for the other tasks here. We therefore selected the bilateral amygdala as a key region of interest, based on the previous literature with a range of emotional tasks (Phan et al., 2002). Thus, the ROI for the evocative images task was made up of two 5 mm radius spheres centred at the MNI coordinates (±22 mm, −4 mm, −12 mm) so as to be robustly in the grey matter of the amygdala as defined functionally by the clusters reported in a previously published ALE meta-analysis of amygdala responsivity (Costafreda et al., 2008).

### MRI data acquisition

All centres operated MRI machines with a main magnetic field of 3 tesla (T). Centres in London and Cambridge operated nominally identical 3T Siemens Tim Trio systems running the syngo MR B17 software with a Siemens 32 channel receive-only phased-array head coil. The Manchester centre operated a 3T Philips Achieva running version 2.6.3.5 software and an eight-element SENSE head coil.

At each visit the imaging session consisted of: localizer scans to set up the positioning of those that would follow; main magnetic field mapping; one run of resting state (360 s); two runs of the monetary incentive delay task (432 s each); two runs of the go/no-go task (262 s each); and two runs of the evocative images task (392 s each).

The tasks were presented to participants in the same order in which they have been covered in this work, namely the two runs of the monetary incentive delay task, followed by the two runs of the go/no-go task, followed by the two runs of the evocative images task. This was so that performance of the monetary incentive delay task would not be adversely affected by a changed emotional state following the presentation of aversive images during the evocative images task.

For each cohort, at the first visit only, a block of structural imaging was performed at the end of the session involving: a high resolution structural scan for anatomical registration and radiological reporting; a proton density scan to provide a second contrast for radiological reporting; and a diffusion tensor imaging sequence for analysis of white matter. The resting state and diffusion tensor data will not be described further here, but will be described elsewhere. Structural images were used in spatial registration, but analysis of structural differences is not described here.

Total in-scanner time was approximately 80 minutes at the first visit, and 60 minutes at all subsequent visits. At every visit, all tasks were practiced outside of the scanner immediately prior to the start of the imaging session.

### Structural acquisition

At London and Cambridge (Siemens), high-resolution T1-weighted volumes were acquired using a magnetization-prepared rapid gradient echo (MPRAGE) sequence (TR=2300 ms, TE=2.98 ms, TI=900 ms, flip angle =9°, field of view =256 mm, image matrix =240×256) with a resolution of 1 mm isotropic. For the volume, 160 abutting straight sagittal slices were collected in an interleaved right to left manner, resulting in whole head coverage. Parallel imaging using Generalized Autocalibrating Partially Parallel Acquisition (GRAPPA) with an acceleration factor of 2 was performed.

At Manchester (Philips), high-resolution T1-weighted volumes were also acquired using an MPRAGE sequence (TR=6.8 ms, TE=3.1 ms, TI=900 ms, flip angle =9°, field of view =270 mm, image matrix =256×256) with an in-plane resolution of 1.055×1.055 mm and a slice thickness of 1.200 mm. For the volume, 126 abutting straight sagittal slices were collected in an interleaved right to left manner, resulting in whole head coverage. Parallel imaging using Sensitivity Encoding (SENSE) with an S reduction of 1.8 was performed.

These T1-weighted volumes followed ADNI protocols (Jack et al., 2008) to minimize inter-centre differences.

### Functional acquisition

At London and Cambridge (Siemens), functional imaging was performed using a multi-echo gradient echo echoplanar imaging (EPI) sequence (TR=2000 ms, TE=13 ms and 31 ms, flip angle =80°, field of view =225 mm, image matrix =64×64) with an in-plane resolution of 3.516×3.516 mm and a slice thickness of 3.000 mm. The phase encoding direction was anterior to posterior. Echo spacing was 0.52 ms. Only the second echo (TE=31 ms) was used in this work.

For each volume, 36 abutting oblique axial slices were collected in an ascending manner at an angle of around 30° to the anterior (AC) and posterior commissure (PC) line. This results in slightly less than whole brain coverage, with the most superior 9 mm not being imaged in most participants.

To achieve the desired resolution and repetition time, parallel imaging using GRAPPA with an acceleration factor of 2 was performed. The first three volumes of each functional run were automatically discarded to allow for T1 saturation effects and are not included in any number of volumes reported here.

At Manchester (Philips) identical parameters were used for EPI acquisition, but with 34 slices being collected and with acceleration achieved using SENSE.

### Data processing

Structural and functional processing was carried out using Analysis of Functional NeuroImages (AFNI) (version AFNI_2011_12_21_1014), FreeSurfer (version freesurfer-x86_64-unknown-linux-gnu-stable5-20130513), Advanced Normalization Tools (ANTs) (version ANTs-1.9.v4-Linux), and FMRIB Software Library’s (FSL) (version 5.0.6) FMRI Expert Analysis Tool (FEAT) (version 6.00). All were run on CentOS 6.5 (version centos-release-6-5.el6.centos.11.2.x86_64).

T1 images were first corrected for intensity non-uniformity (AFNI’s 3dUniformize) before having extracerebral tissues removed (as part of FreeSurfer’s recon-all pipeline). The whole brain images were then non-linearly registered to the MNI ICBM152 non-linear 6th generation symmetric average brain stereotaxic registration model in a 2 mm isotropic voxel space (ANTs’ antsRegistration).

EPIs were corrected for slice timing effects (AFNI’s 3dTshift) before each volume was registered (AFNI’s 3dvolreg) to the volume most similar, in the least squares sense, to all others (in-house code). For each task a summary of movement was recorded as the speed of motion over the runs (i.e. the sum of framewise displacements (FD) over the time taken for the runs, measured in mm/s).

The residual extracerebral tissues were then removed using FSL’s Brain Extraction Tool (BET). Linear registration to the T1 image was achieved through a Boundary Based Registration (BBR) approach (FSL’s epi_reg) before combining transformations to bring the EPIs into the same standard stereotaxic space as the transformed T1 (ANTs’ antsApplyTransforms). Finally, these were smoothed with a three-dimensional Gaussian kernel of full width at half maximum of 6.0 mm (i.e. standard deviation =2.5 mm) (AFNI’s 3dBlurInMask).

### fMRI task modelling

Task processing and modelling was carried out using E-Prime (version 2.0.8.90), Microsoft Office Excel 2007 (version 12.0.4518.1014), in-house Python (version 2.7.6) scripts, and FSL.

Data from task responses were processed into usable formats (E-Prime’s E-DataAid) before behavioural data and timings were extracted (Excel) and processed further (Python scripts) into three-column-format text files for each event type for compatibility with FEAT.

FMRIB’s Improved Linear Modelling (FILM) prewhitening was performed on all voxel time courses. Estimates of six motion parameters (translations in the three orthogonal directions along with pitch, roll and yaw) calculated during preprocessing (AFNI’s 3dvolreg) were included in each model as confounding explanatory variables.

In all models convolution with a haemodynamic response function (HRF) was performed, this being FSL’s commonly used gamma function with standard deviation 3 s and mean lag 6 s. No temporal derivatives were used in any model. All models had the same temporal filtering applied to them as was done to the image data.

### Monetary incentive delay task

Nine explanatory variables were used for modelling the task itself. These were the three different general conditions – reward, neutral or loss – with each of these having three potential phases – anticipation, successful outcome or unsuccessful outcome. ‘Anticipation’ was modelled as a block beginning at the cue (an arrow or line) onset and ending at the trial (a star) onset (these blocks lasting between approximately 3 s and 5 s – that is, the combined time of the cue and blank screen before the star). ‘Outcome’ was modelled as an immediately abutting block beginning at the trial (a star) onset and ending two seconds later. A high-pass filter cut-off of periods above 50 s was applied to both the data and the model. The contrast further explored in this work is that of ‘reward anticipation’ compared with ‘neutral anticipation’, with ‘reward anticipation’ being expected to show greater BOLD response (Knutson et al., 2001).

### Go/no-go task

Two explanatory variables were used for modelling the task itself, one for ‘successful no-go’ and the other for ‘unsuccessful no-go’. These were modelled against an implicit baseline of ‘go’. Both ‘successful no-go’ and ‘unsuccessful no-go’ were modelled as events lasting 0.1 s. A high-pass filter cut-off of periods above 120 s was applied to both the data and the model. The contrast further explored in this work is that of ‘successful no-go’ compared with the implicit baseline of ‘go’, with ‘successful no-go’ being expected to show greater BOLD response (Garavan et al., 2003).

### Evocative images task

Two explanatory variables were used for modelling the task itself, one for ‘aversive’ images and the other for ‘neutral’ images. Both ‘aversive’ and ‘neutral’ were modelled as blocks lasting 32.4 s. A high-pass filter cut-off of periods above 100 s was applied to both the data and the model. The contrast further explored in this work is that of ‘aversive’ compared with ‘neutral’ images, with ‘aversive’ images being expected to show greater BOLD response (Asensio et al., 2010).

### Higher level analysis

FEAT was used to run all the models discussed above within a general linear model framework. As each task was run twice in each imaging session the mean of the results for both runs (at the individual level) was used in all higher level analyses.

This voxelwise analysis was extended to a group level in a mixed-effects analysis using FSL’s FLAME 1 (one-sample *t*-test) controlling for centre, age and sex. In calculating the whole brain group maps as part of the task characterization investigation, data from the baseline (i.e. neither a drug nor placebo) session of the 43 inter-centre participants were used (a between-centre factor was included in the model). The *Z* statistic images shown in this work for the evocative and go/no-go tasks were thresholded using clusters determined by *Z*>3.1 (i.e. an initial uncorrected cluster forming threshold of *p*<0.001) and a (corrected) cluster significance threshold of *p*<0.05. These initial cluster thresholds are higher than those commonly seen, and follow the advice given by Woo et al. (2014) relating to minimum valid thresholds. The equivalent images for the monetary incentive delay task were thresholded using clusters determined by *Z*>4.5 and a (corrected) cluster significance threshold of *p*<0.05. This initial cluster threshold was raised compared with the other tasks due to the relatively stronger response expected in comparison to the other tasks, so that clusters would still be able to form and be interpretable. This group analysis was performed on the whole brain, insofar as including all those voxels which all participants had in common (areas outside this common coverage are shown masked in figures).

For the tasks which have temporal characteristics similar to block designs (monetary incentive delay and evocation) the contrasts’ mean percentage signal changes within their ROIs were calculated (FSL’s Featquery), while for the fast event-related design (go/no-go) arbitrary units based on the parameter estimates were used, as percentage signal change is not usefully interpretable in this case.

### Inter-centre differences

Non-image statistical analysis was carried out using IBM SPSS Statistics (version 22.0). When appropriate, values are given as mean±standard deviation.

Between centre differences were tested for using one-way analysis of variance (ANOVA). When significant differences were found between centres Tukey’s honestly significant difference (HSD) was used as the post-hoc test. Heterogeneity of variance was examined using Levene’s test, and if found to be significant (*p*<0.05) Welch’s F was used. Post-hoc testing for data not meeting the homogeneity of variance assumption was carried out using the Games Howell method. Normality of data from each centre was tested using the Shapiro–Wilk method, and, if found to be significantly (*p*<0.05) skewed, a non-parametric Kruskal–Wallis test performed in place of an ANOVA. Post-hoc tests for data examined using a non-parametric approach were carried out using the Mann–Whitney U test. All reported *p* values are those before any correction for either the number of tasks, or the number of tests carried out on the behavioural and summary imaging measures of those tasks, but they have been corrected for the number of post-hoc tests carried out for a particular measure.

FEAT was used to perform a voxelwise ANOVA, examining between-centre differences to produce F statistic images of the whole brain for each task.

## Results

### Participants

A summary of the groupings and participant information is given in Table 1. No differences were found between centres for age, sex, or handedness, consistent with the matching process.

**Table 1.** Participant information.

	London	Cambridge	Manchester	ANOVA/χ^2^	Combined
	(*n*=15)	(*n*=15)	(*n*=13)	(*n*=15, 15, 13)	(*n*=43)
Age (years)	40.5±8.5 (21–53)	37.9±9.3 (22–52)	41.0±9.3 (25–56)	*F*_2,40_=0.50, *p*=0.61	39.7±8.9 (21–56)
# female	3	3	3	χ^2^(2, *N*=43)=0.05, *p*=0.97	9
# left handed /ambidextrous	4/1	4/1	0/2	χ^2^(4, *N*=43)=4.61, *p*=0.33	8/4

Each of the three tasks – monetary incentive delay, go/no-go and evocative images – will be fully covered in turn, with each broken down into its meta-analysis/ROI definition, task characterization, and inter-centre differences.

### Monetary incentive delay – ALE meta-analysis

**Figure 1.**
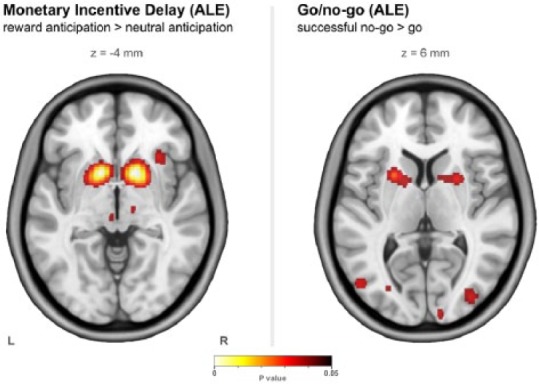
Clusters found through the activation likelihood estimation (ALE) meta-analyses. ALE was performed for each task with a false discovery rate (FDR) of *p*<0.05 (corrected) and a minimum cluster volume of 0.6 ml.

For the monetary incentive delay task, we identified an initial total of 487 studies from searches on PubMed, and 170 from the BrainMap database, with 156 of the latter being duplicates of the former. This left a total of 501 studies. After abstract screening (501 studies) and full-text review (90 studies), 17 studies remained, representing 292 healthy participants with a total of 170 activation foci, shown in Table 2. Four clusters were found after carrying out the ALE analysis, the two largest of these being focused on the anterior region of the left and right putamen and overlapping with portions of caudate, nucleus accumbens and globus pallidus (all bilaterally). All clusters found through ALE analysis are listed in Supplementary Table 1 and are shown in Figure 1. The ROI for this task was made up of bilateral 5 mm radius spheres centred at the co-ordinates ((L–R, P–A, I–S) in MNI space) (±14 mm, 12 mm, −4 mm); that is, striatum (dorsal putamen/caudate).

**Table 2.** Studies included in the monetary incentive delay ALE meta-analysis.

Year	Author	Participants	Foci	Design	Scanner strength (T)	Whole brain analysis
2003	Knutson et al.	12	10	Knutson	1.5	No
2004	Knutson et al.	8	8	Knutson	3	Yes
2006	Juckel et al.	10	9	Knutson	1.5	No
2007a	Wrase et al.	14	18	Knutson	1.5	No
2007b	Wrase et al.	16	2	Knutson	1.5	No
2008	Knutson et al.	12	8	Knutson	1.5	Yes
2008	Schlagenhauf et al.	10	12	Knutson	1.5	No
2008	Schmack et al.	44	2	Knutson	1.5	No
2008	Strohle et al.	10	7	Knutson	1.5	No
2009	Beck et al.	19	6	Knutson	1.5	No
2010	Bjork et al.	24	10	Bjork	3	No
2011	de Greck et al.	20	12	Knutson	1.5	Yes
2012	Balodis et al.	14	7	Knutson	3	No
2013	Cho et al.	30	18	Knutson	3	Yes
2012	Enzi et al.	19	15	Knutson	1.5	Yes
2013	Edel et al.	12	4	Knutson	1.5	No
2013	Saji et al.	18	22	Knutson	1.5	Yes
	**Total**	**292**	**170**			

### Monetary incentive delay – task characterization

Accuracy was not found to be different between the three types of trial (reward, neutral, and loss) (F_2,126_=1.33, *p*=0.27). Response time did differ (F_2,126_=4.31, *p*=0.015), with post-hoc analysis showing that response time of neutral trials was slower than loss trials (*p*=0.003) with no other differences apparent. Supplementary Table 6 lists behavioural results.

**Figure 2.**
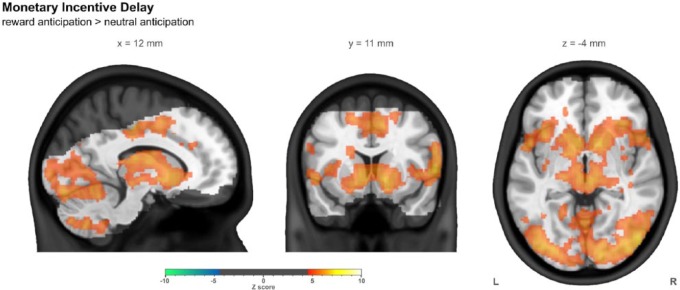
The contrast of reward anticipation with neutral anticipation in the monetary incentive delay task in the combined group (*n*=43), controlling for centre, age and sex. Images were thresholded using clusters determined by *Z*>4.5 and a (corrected) cluster significance threshold of *p*<0.05. The slices shown were chosen such that all three intersect with the left side of the ROI used later in this work. The greyed out portion shows areas outside common coverage.

The strongest observed response to reward anticipation (in terms of Z statistics) was in the primary visual cortex, with other strong responses in the caudate and anterior insula bilaterally. A spatially widespread response was observed in other visual areas and a large group of regions incorporating the striatum, thalamus and insula, along with the supplementary motor area. No regions were seen to have a stronger response to neutral anticipation. Whole brain summary images of the reward anticipation > neutral anticipation contrast are shown in Figure 2, while more detailed images are shown in Supplementary Figure 1. Supplementary Table 3 lists the locations of clusters larger than 2 ml.

For this contrast, in the striatal ROI, the mean response (*n*=43) was 0.53%±0.05% with a mean *Z* statistic of 5.84±0.31. Supplementary Table 6 lists ROI results.

### Monetary incentive delay – inter-centre differences

In the monetary incentive delay task the accuracy of loss trials was different between the centres (Kruskal–Wallis, *p*=0.006), with Manchester having lower accuracy than Cambridge (Mann–Whitney, *p*=0.007). The response time of successful loss trials was different between the centres (Kruskal–Wallis, *p*=0.004), with Manchester being slower than Cambridge (Mann–Whitney, *p*=0.012). Three of the other measures for the monetary incentive delay task – amount won, reward accuracy and neutral accuracy – had skewed distributions (Shapiro–Wilk test) and so a non-parametric (Kruskal–Wallis) test was performed (*p*=0.01, 0.04 and 0.03, respectively). These do not survive at the α=0.05 level after a Bonferroni correction for the number of tests performed on the behavioural measures of this task (approximately seven independent tests), but are reported here for completeness. Appropriately corrected Mann–Whitney U post-hoc tests reveal that Manchester participants won less than those in London (*p*=0.009), and had lower accuracy at reward trials than those in London (*p*=0.021).

**Figure 3.**
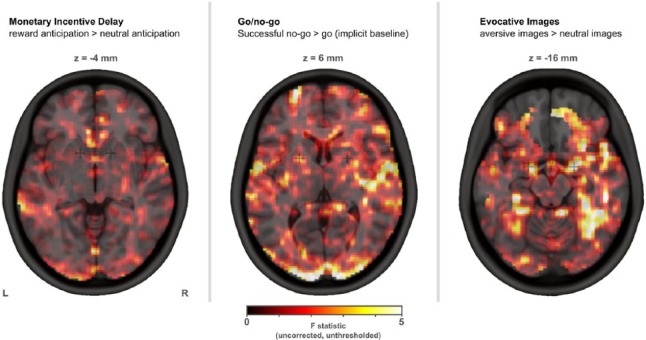
Unthresholded F maps exploring inter-centre differences. No significant imaging differences were found between centres at this whole brain (voxelwise) level.

No imaging differences were found between centres at the whole brain (voxelwise) level. Unthresholded F maps are shown below in Figure 3.

No differences were found between centres with regard to the ROI results.

### Go/no-go – ALE meta-analysis

For the go/no-go task, we identified an initial total of 353 studies from searches on PubMed, and 94 from the BrainMap database, with 80 of the latter being duplicates of the former. This left a total of 367 studies. After abstract screening (367 studies) and full-text review (189 studies), 12 studies remained, representing 243 healthy participants with a total of 180 activation foci, shown in Table 3. 12 clusters were found after carrying out the ALE analysis, distributed around the brain, but with a concentration around the striatum. All clusters found through ALE analysis are listed in Supplementary Table 2 and are shown in Figure 1. The ROI for this task was made up of bilateral 5 mm radius spheres centred at the co-ordinates (±22 mm, 8 mm, 6 mm); that is, striatum (dorsal putamen).

**Table 3.** Studies included in the Go/no-go ALE meta-analysis.

Year	Author	Participants	Foci	Design	Scanner strength (T)	Whole brain analysis
2002	Garavan et al.	14	16	X/Y Alternating	1.5	Yes
2003	Garavan et al.	16	7	X/Y Alternating	1.5	Yes
2004	Hester et al.	15	21	X/Y Alternating	1.5	Yes
2004	Kelly et al.	15	23	X/Y Alternating	1.5	Yes
2005	Maltby et al.	11	5	X is Go, K is No-go	1.5	Yes
2007	Epstein et al.	9	15	Multiple Go Cues, X is No-go	1.5	Yes
2009	Welander-Vatn et al.	28	12	Multiple Go Cues, V is No-go	1.5	Yes
2012	Bannbers et al.	14	2	X/Y Alternating	3	Yes
2012	Sebastian et al.	24	19	Multiple Go Cues, X is No-go	3	Yes
2013a	Sebastian et al.	49	26	Multiple Go Cues, X is No-go	3	Yes
2013b	Sebastian et al.	24	25	Multiple Go Cues, X is No-go	3	Yes
2013	van der Salm et al.	24	9	X/Y Alternating	3	Yes
	**Total**	**243**	**180**			

### Go/no-go – task characterization

Response time was found to be different between successful ‘go’ and unsuccessful ‘no-go’, with faster button presses for unsuccessful ‘no-go’ (*t*=6.69, *p*<0.0001, df=42). Supplementary Table 7 lists behavioural results.

**Figure 4.**
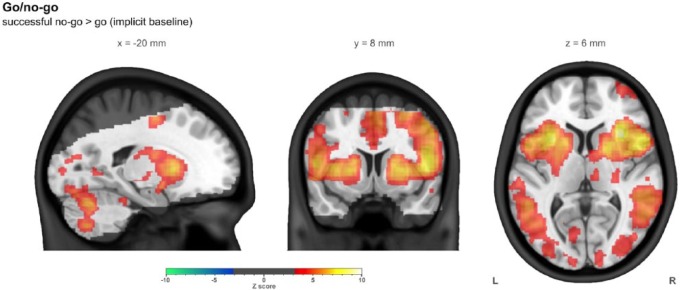
The contrast of successful no-go with go (implicit baseline) in the go/no-go task in the combined group (*n*=43), controlling for centre, age and sex. Images were thresholded using clusters determined by *Z*>3.1 and a (corrected) cluster significance threshold of *p*<0.05. The slices shown were chosen such that all three intersect with the left side of the ROI used later in this work. The greyed out portion shows areas outside common coverage.

The strongest observed response to successful ‘no-go’ (in terms of *Z* statistics) was in the anterior insula bilaterally, with other strong responses in right inferior frontal gyrus, putamen and thalamus. A spatially widespread response was observed across the brain, including right dorsolateral prefrontal cortex and bilateral supplementary motor area. Only ventromedial prefrontal cortex was observed to have greater response to ‘go’ (implicit baseline). Whole brain summary images of the successful no-go>go (implicit baseline) contrast are shown in Figure 4, while more detailed images are shown in Supplementary Figure 2. Supplementary Table 4 lists the locations of clusters larger than 2 ml.

For this contrast, in the striatal ROI, the mean response (*n*=43) was 0.36 arbitrary units ±0.06, with a mean *Z* statistic of 6.27±0.47. Supplementary Table 7 lists ROI results.

### Go/no-no – inter-centre differences

No behavioural differences were found between centres for the go/no-go task.

No imaging differences were found between centres at the whole brain (voxelwise) level. Unthresholded F maps are shown below in Figure 3.

No differences were found between centres with regard to the ROI results.

### Evocative images – task characterization

Although the range of response times was large (280ש1373 ms for neutral images and 267–1651 for aversive images) there was a very strong correlation between the two times (*r*=0.94, *p*<0.00001, df=58). The difference in response time (21 ms) was not significant between the aversive and neutral images (*t*=1.86, *p*=0.068, df=42). Supplementary Table 8 lists behavioural results.

**Figure 5.**
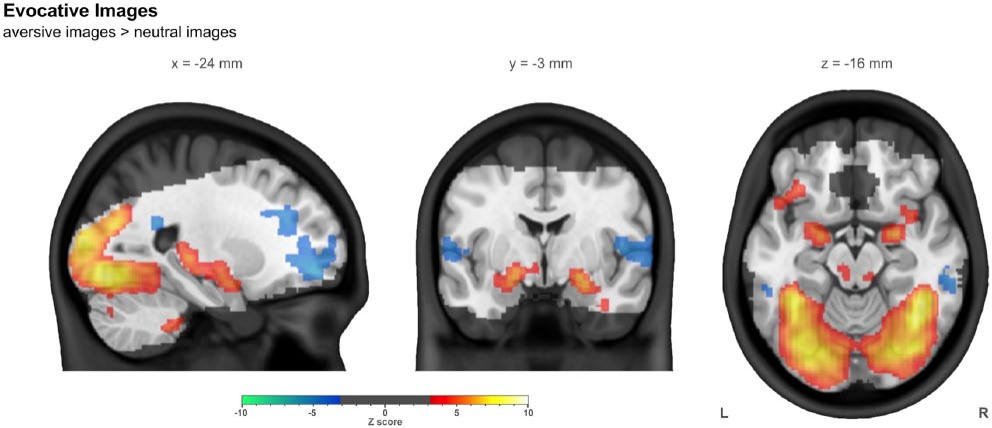
The contrast of aversive images with neutral images in the evocative images task in the combined group (*n*=43), controlling for centre, age, and sex. Images were thresholded using clusters determined by *Z*>3.1 and a (corrected) cluster significance threshold of *p*<0.05. The slices shown were chosen such that all three intersect with the left side of the ROI used later in this work. The greyed out portion shows areas outside common coverage.

The strongest response observed to aversive images (in terms of *Z* statistics) was in visual cortex, with the strongest response outside of this region being in the amygdala bilaterally. Strong response was also observed in thalamus and medial hippocampus. Greater response to neutral images was observed in prefrontal and auditory cortices. Whole brain summary images of the aversive images > neutral images contrast are shown in Figure 5, while more detailed images are shown in Supplementary Figure 3. Supplementary Table 5 lists the locations of clusters larger than 2 ml.

For this contrast, in the amygdala ROI, the mean response (*n*=43) was 0.32%±0.09%, with a mean *Z* statistic of 3.90±1.06. Supplementary Table 8 lists ROI results.

Rate of motion (i.e. mm/s) was not found to differ significantly for different tasks (F_2,126_=2.61, *p*=0.08).

### Evocative images – inter-centre differences

No behavioural differences were found between centres for the evocative images task.

No imaging differences were found between centres at the whole brain (voxelwise) level. Unthresholded F maps are shown in Figure 3.

In the evocative images task, mean response (percentage signal change) within the amygdala ROI was found to differ between the centres (*F*_2,40_=5.06, *p*=0.01), along with, as one would expect given the signal change, the mean *Z* statistic in this region (*F*_2,40_=5.98, *p*=0.005). This was due to the Manchester participants having a lower response for this aversive images > neutral images contrast.

## Discussion

We report here the establishment of an fMRI platform, ICCAM, to study mechanisms of relevance to relapse in addiction. Across three tasks investigating reward sensitivity, inhibitory control and emotional reactivity, we have examined their characteristics, and inter-centre differences for behavioural, whole brain and ROI measures. This study raised a number of issues, which we now discuss in turn.

Importantly our three tasks resulted in the expected pattern of brain responses consistent with existing evidence. Thus the monetary incentive delay task resulted in responses in regions such as the visual cortex, striatum, prefrontal and insula cortices consistent with previous studies (Knutson et al., 2001). The influence of variations in the task on the patterns of brain responses have been described elsewhere (Hommer et al., 2011; Limbrick-Oldfield et al., 2013). Though many people use the monetary incentive delay task, most adapt it to some extent so that it is no longer a standardized task. For instance, the ICCAM version of the monetary incentive delay task prioritized imaging ‘anticipation of reward’ since this primary contrast has been found altered in addiction and is of relevance to relapse. Therefore we were less interested in brain responses to loss or outcomes.

The pattern of brain response elicited by our monetary incentive delay task was consistent with that derived from the meta-analysis. Many fMRI studies of the monetary incentive delay task used spatially constrained approaches –that is, analyses performed within ROIs of varying size, focused on striatal regions. Out of the 17 studies used here, 11 were not ‘whole brain’ analyses. Indeed, in the original fMRI monetary incentive delay study (Knutson et al., 2001), a limited acquisition of coronal slices was used, limiting coverage to a block including the striatum, and our own coverage is itself limited, as can be seen throughout the figures (such as Figure 2). By comparison, of the 12 go/no-go studies included in the meta-analysis, none used such a spatially constrained approach.

The responses to the go/no-go task in inferior frontal gyrus, striatum, insula and thalamus were consistent with previous studies (Luijten et al., 2014; Steele et al., 2013). Our meta-analysis of similar go/no-go tasks resulted in a striatal ROI, though this was more dorsal than the one derived from the meta-analysis of the monetary incentive delay task. This association between ventral striatum associated with reward processing and dorsal striatum with habit or compulsive behaviours, and the importance of fronto-corticostriatal loops in inhibitory control, have been well documented (Everitt and Robbins, 2005; Koob and Volkow, 2010).

Although there is often a focus on the inferior frontal gyrus (IFG) when discussing go/no-go tasks, this did not emerge in our ALE meta-analysis (which closely follow the results presented in the task characterization here). IFG response was observed in our task, though was weaker than insular or striatal responses. This might be explained by the ‘simple’ design task used here (and thus the strict criteria in our meta-analysis), while the majority of those in the literature used more complex designs (Criaud and Boulinguez, 2013). In the extensive ALE meta-analysis performed by Criaud and Boulinguez (2013) examining several facets of fMRI go/no-go tasks, it is also suggested that typical no-go activity is mostly driven by attention, not inhibition, though this is still a current topic of debate (Aron et al., 2014).

Both the go/no-go and monetary incentive delay task resulted in robust responses in the insula, particularly anterior insula. This brain region has been shown to be involved in self-regulation and reward seeking, as well as in emotional awareness, through integrating sensory information into cognitive, affective and physiological processes, along with being part of a task general network (Gu et al., 2013; Menon and Uddin, 2010; Nelson et al., 2010). With regard to addiction, the insula appears also to be involved with critical functions such as craving, and the landmark description that damage to its structure substantially increased the likelihood of smoking cessation (Garavan, 2010; Naqvi and Bechara, 2009).

The evocative images task produced a robust response in the dorsal amygdala, along with inferior portions of the globus pallidus, with the highest response near the amygdala overlapping with the predetermined amygdala ROI. Such a pattern is consistent with previous studies using an evocative task or one that requires emotional processing (Costafreda et al., 2008; Sergerie et al., 2008). We were particularly interested in demonstrating a robust response in the amygdala, since dysregulation in this region is implicated in relapse vulnerability in addiction, in particular those involving stress (Koob et al., 2014).

Even though this comparison between centres did not utilize a travelling participants design, such as those of Friedman et al. (2008), Gee et al. (2015) and Suckling et al. (2012), it demonstrates that different groups of participants at different centres produce markedly similar patterns of response to the tasks in our ICCAM platform. Recent explorations of both functional and structural neuroimages acquired from multiple centres have unequivocally demonstrated high levels of within- and between-centre reliability, as well as small between-centre variances relative to the total variance (Gee et al., 2015; Suckling et al., 2012).

However, the lack of significant whole brain differences in the participants examined here does not necessarily imply that with larger groupings and different patient populations there would not be differences observed.

Although much effort was made to run the study in as similar a manner possible at each centre, there were inevitably slight differences between the experimental set-ups which may have driven differences. In the monetary incentive delay task a lower accuracy led to slightly less money being won by the Manchester group, though brain response wasn’t observed to be different. In the evocative images task a centre difference was found in the amygdala ROI (Supplementary Table 8) and was driven by the Manchester group (at the whole brain level, no significant differences were observed). One factor may have been the means by which images were projected, which was almost identical in London and Cambridge but differed in Manchester, where images were projected in a different manner, creating a less bright and so possibly less salient image, creating a smaller difference in activity between the aversive and neutral images.

Although in this analysis we have explored differences between centres, in the patient study itself participants were recruited so that there would be a roughly equal proportion of cases to controls at each centre. Centre was used as a covariate in the characterization of the tasks. Thus, although small differences were observed in the monetary incentive delay task behavioural results, and the evocative images task neuroimaging results, these may be regarded as effects of centre (which is used as a covariate in all analyses).

## Conclusion

We have demonstrated here the establishment of an fMRI platform involving three different tasks, repeated at multiple sessions and at three different centres. The establishment of this platform was critical to provide a framework to explore three key processes in the neurobiology of relapse vulnerability in addiction: reward, inhibitory control and emotional regulation. This allows for an evidence base to inform future development in treatment to be provided within reasonable time periods. Future papers will present the results of these tasks in our healthy and patient groups, and under pharmacological modulation.

## Supplementary Material

Supplementary material
