# Supplementary material for: The ICCAM platform study: An experimental medicine platform for evaluating new drugs for relapse prevention in addiction. Part B: fMRI description
Source: J Psychopharmacol. 2016 Oct 4;31(1):3–16. doi: 10.1177/0269881116668592 (PMC5367542; doi:10.1177/0269881116668592)
Supplement: Supplementary material [file JOP_668592_Supplementary_table.pdf]

## SUPPLEMENTARY MATERIALS

### *SUPPLEMENTARY TABLE 1.*

| *Monetary incentive delay: reward anticipation > neutral anticipation<br>17 experiments, 296 subjects* | | | | |
|---|---|---|---|---|
| Region | Cluster volume (ml) | Weighted centre MNI coordinates (mm) | | |
| | | x | y | z |
| Right Putamen | 7.60 | 15 | 9 | -4 |
| Left Putamen | 6.91 | -15 | 9 | -6 |
| Right Anterior Insula | 0.94 | 33 | 23 | -4 |
| Left Inferior Frontal Precentral Gyrus | 0.94 | -50 | 9 | 28 |

Clusters are named according to the structure with the highest probability at that position in the Harvard-Oxford Cortical and Subcortical Structural Atlases.

*SUPPLEMENTARY TABLE 2.*

*Go/no-go: successful no-go > go*
*12 experiments, 246 subjects*

| Region | Cluster volume (ml) | Weighted centre MNI coordinates (mm) | | |
|---|---|---|---|---|
| | | x | y | z |
| Right Frontal Pole | 2.77 | 35 | 40 | 24 |
| Left Putamen | 2.20 | -20 | 7 | 4 |
| Right Supplementary Motor Area | 1.73 | 1 | -1 | 63 |
| Supramarginal Gyrus | 1.49 | 49 | -38 | 44 |
| Superior Temporal Gyrus | 1.34 | 56 | -27 | -2 |
| Right Occipital Pole | 1.33 | 30 | -90 | 9 |
| Precentral Gyrus | 1.22 | -45 | -4 | 50 |
| Right Lateral Occipital Cortex | 1.20 | 31 | -62 | 52 |
| Right Putamen | 0.82 | 21 | 6 | 6 |
| Left Lateral Occipital Cortex | 0.74 | -50 | -75 | 3 |
| Right Lateral Occipital Cortex | 0.70 | 48 | -72 | -2 |
| Left Lateral Occipital Cortex | 0.66 | -22 | -63 | 54 |

Clusters are named according to the structure with the highest probability at that position in the Harvard-Oxford Cortical and Subcortical Structural Atlases.

*SUPPLEMENTARY FIGURE 1.*
A detailed view of the contrast shown in Figure 2. The contrast of reward anticipation with neutral anticipation in the monetary incentive delay task in the combined group (n = 43), controlling for centre, age, and sex. Images were thresholded using clusters determined by Z > 4.5 and a (corrected) cluster significance threshold of $p < 0.05$. The greyed out portion shows areas outside common coverage.

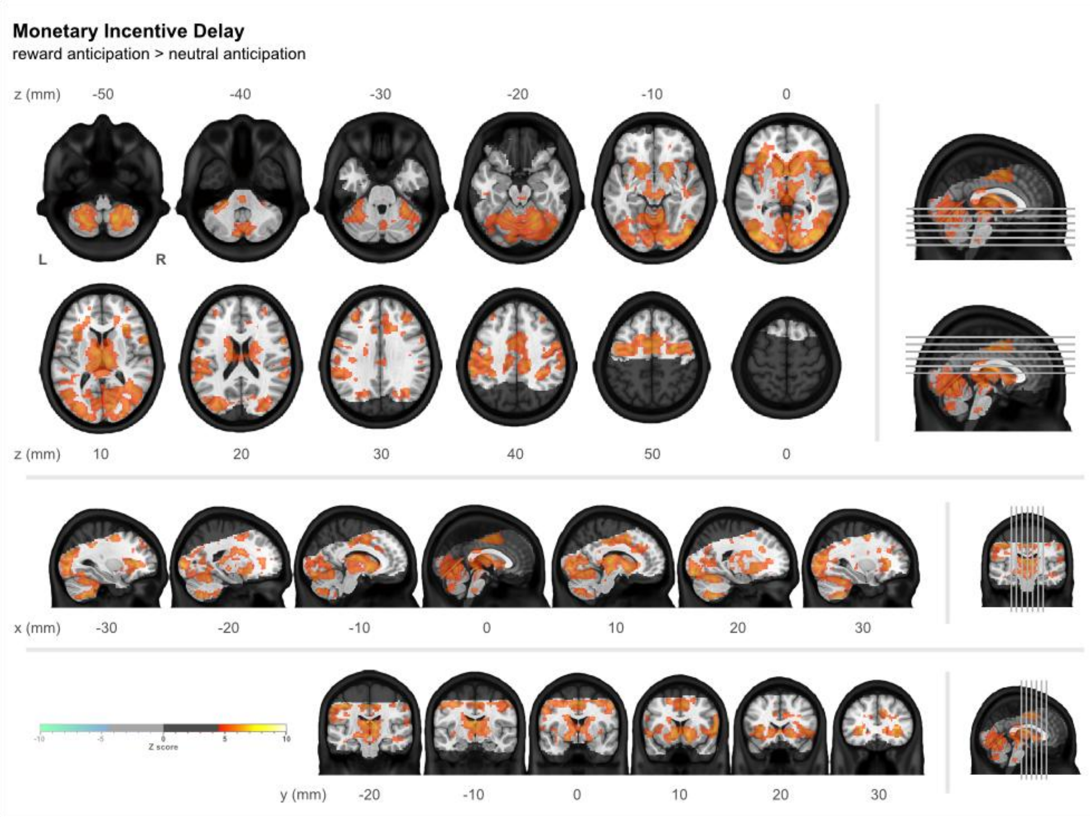

*SUPPLEMENTARY TABLE 3.*

*Monetary incentive delay: reward anticipation > neutral anticipation*

| Region | Cluster volume (ml) | p | Local maxima Z score | MNI coordinates of maxima (mm) | | |
|---|---|---|---|---|---|---|
| | | | | x | y | z |
| Cluster 1 (Occipital Lobe) | 185 | < 0.0001 | | | | |
| Lateral Occipital Cortex, inferior division | | | 7.05 | 48 | -78 | -6 |
| Lingual Gyrus | | | 6.97 | -48 | -66 | 4 |
| Occipital Pole | | | 6.94 | -18 | -98 | 2 |
| Occipital Fusiform Gyrus | | | 6.92 | 18 | -90 | -10 |
| Occipital Pole | | | 6.57 | 18 | -94 | -14 |
| Cerebellum | | | 6.55 | 18 | -58 | -52 |
| Cluster 2 (Frontal Lobe) | 175 | < 0.0001 | | | | |
| Inferior Frontal Gyrus, pars opercularis | | | 6.92 | 58 | 12 | 6 |
| Frontal Operculum Cortex | | | 6.92 | 32 | 30 | 8 |
| Frontal Orbital Cortex | | | 6.60 | 30 | 32 | 2 |
| Supramarginal Gyrus, anterior division | | | 6.60 | 56 | -32 | 36 |
| Cingulate Gyrus, anterior division | | | 6.59 | -28 | 30 | 4 |
| Thalamus | | | 6.58 | 8 | -14 | 16 |
| Cluster 3 (Frontal Lobe) | 2.76 | < 0.0001 | | | | |
| Paracingulate Gyrus | | | 5.87 | -34 | 40 | 34 |
| Superior Frontal Gyrus | | | 5.14 | -32 | 38 | 44 |
| Paracingulate Gyrus | | | 4.83 | -34 | 50 | 18 |
| Cluster 4 (Parietal Lobe) | 2.08 | < 0.0001 | | | | |
| Postcentral Gyrus | | | 5.68 | 32 | -38 | 42 |
| Postcentral Gyrus | | | 5.66 | 38 | -38 | 42 |
| Postcentral Gyrus | | | 4.84 | 42 | -26 | 38 |

Coordinates (in MNI space) and Z score maxima for cluster-based statistical contrasts (all $Z > 4.5$, $p < 0.05$). Only clusters larger than 2 ml are shown. Clusters are first named by their general position according to the Talairach Daemon, while maxima are named according to the structure with the highest probability at that position in the Harvard-Oxford Cortical and Subcortical Structural Atlases.

*SUPPLEMENTARY FIGURE 2.*

A detailed view of the contrast shown in Figure 3. The contrast of successful no-go with go (implicit baseline) in the go/no-go task in the combined group (n = 43), controlling for centre, age, and sex. Images were thresholded using clusters determined by Z > 3.1 and a (corrected) cluster significance threshold of $p < 0.05$. The greyed out portion shows areas outside common coverage.

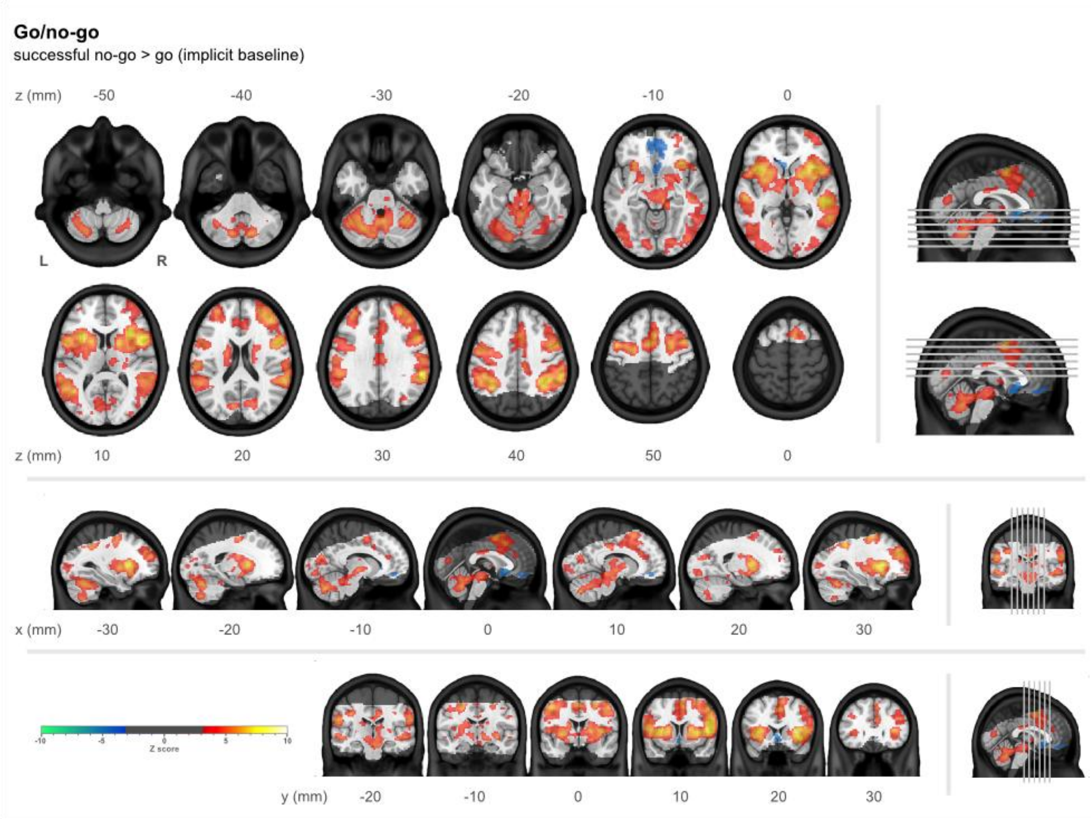

*SUPPLEMENTARY TABLE 4.*

| *Go/no-go: successful no-go > go (implicit baseline)* | | | | | | |
|---|---|---|---|---|---|---|
| Region | Cluster volume (ml) | p | Local maxima Z score | MNI coordinates of maxima (mm) | | |
| | | | | x | y | z |
| Cluster 1 (Sub-lobar) | 371 | < 0.0001 | | | | |
| Insular Cortex | | | 9.34 | 32 | 18 | 6 |
| Subcallosal Cortex | | | 8.38 | -32 | 14 | 8 |
| Supramarginal Gyrus, posterior division | | | 8.34 | 60 | -44 | 26 |
| Superior Temporal Gyrus, posterior division | | | 8.23 | 48 | -28 | -2 |
| Inferior Frontal Gyrus, pars opercularis | | | 8.02 | 48 | 8 | 10 |
| Precentral Gyrus | | | 7.81 | 50 | 8 | 6 |
| Cluster 2 (Frontal Lobe) | 9.2 | < 0.0001 | | | | |
| Paracingulate Gyrus | | | 5.97 | -34 | 46 | 28 |
| Paracingulate Gyrus | | | 5.36 | -30 | 54 | 20 |
| Frontal Pole | | | 5.20 | -38 | 56 | 14 |
| Paracingulate Gyrus | | | 4.54 | -36 | 46 | 16 |
| Cluster 3 (Limbic Lobe) | 3.096 | 0.0001 | | | | |
| Frontal Medial Cortex | | | -4.48 | -10 | 50 | -12 |
| Frontal Medial Cortex | | | -4.42 | 12 | 46 | -10 |
| Frontal Medial Cortex | | | -4.21 | 4 | 48 | -14 |
| Paracingulate Gyrus | | | -4.14 | 6 | 36 | -12 |
| Frontal Medial Cortex | | | -3.99 | -2 | 54 | -8 |
| Subcallosal Cortex | | | -3.40 | -8 | 30 | -8 |

Coordinates (in MNI space) and Z score maxima for cluster-based statistical contrasts (all $Z > 3.1$, $p < 0.05$). Only clusters larger than 2 ml are shown. Clusters are first named by their general position according to the Talairach Daemon, while maxima are named according to the structure with the highest probability at that position in the Harvard-Oxford Cortical and Subcortical Structural Atlases.

*SUPPLEMENTARY FIGURE 3.*

A detailed view of the contrast shown in Figure 4. The contrast of aversive images with neutral images in the evocative images task in the combined group (n = 43), controlling for centre, age, and sex. Images were thresholded using clusters determined by Z > 3.1 and a (corrected) cluster significance threshold of $p < 0.05$. The greyed out portion shows areas outside common coverage.

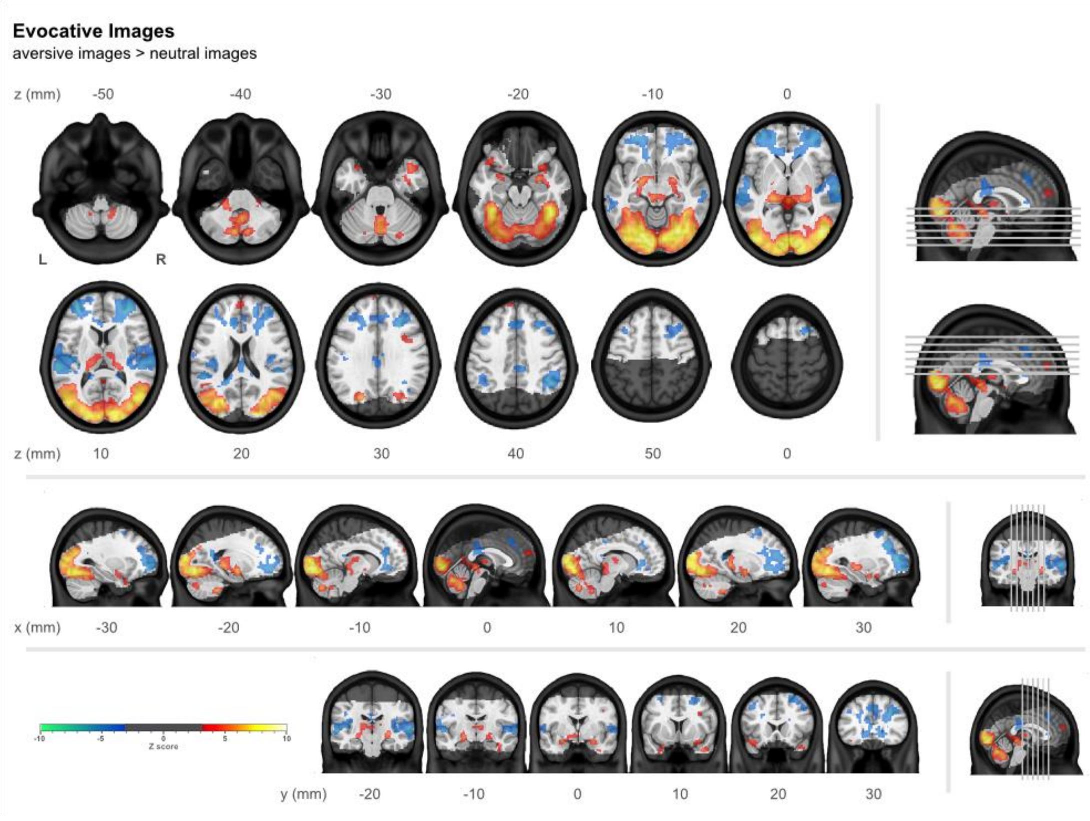

*SUPPLEMENTARY TABLE 5.*

| *Evocative images: aversive images > neutral images* | | | | | | |
|---|---|---|---|---|---|---|
| Region | Cluster volume (ml) | p | Local maxima Z score | MNI coordinates of maxima (mm) | | |
| | | | | x | y | z |
| Cluster 1 (Occipital Lobe) | 182 | < 0.0001 | | | | |
| Lateral Occipital Cortex, inferior division | | | 8.83 | 48 | -74 | -2 |
| Occipital Fusiform Gyrus | | | 8.81 | 30 | -78 | -12 |
| Lateral Occipital Cortex, inferior division | | | 8.81 | 52 | -70 | 6 |
| Occipital Pole | | | 8.74 | 18 | -94 | 18 |
| Lingual Gyrus | | | 8.72 | -20 | -84 | -10 |
| Occipital Pole | | | 8.69 | -4 | -92 | -6 |
| Cluster 2 (Frontal Lobe) | 69.8 | < 0.0001 | | | | |
| Frontal Pole | | | -6.71 | 34 | 50 | 10 |
| Paracingulate Gyrus | | | -6.13 | -34 | 54 | 8 |
| Frontal Medial Cortex | | | -5.73 | -26 | 52 | -8 |
| Middle Frontal Gyrus | | | -5.67 | 30 | 28 | 32 |
| Paracingulate Gyrus, | | | -5.60 | -32 | 46 | 8 |
| Left Cerebral White Matter | | | -5.54 | 22 | 40 | -4 |
| Cluster 3 (Sub-lobar) | 28.5 | < 0.0001 | | | | |
| Amygdala | | | 6.36 | -20 | -6 | -12 |
| Thalamus | | | 6.22 | -20 | -30 | 0 |
| Brain-Stem | | | 6.17 | -6 | -30 | -6 |
| Amygdala | | | 6.01 | 24 | -4 | -14 |
| Thalamus | | | 5.77 | 22 | -30 | 0 |
| Brain-Stem | | | 5.65 | 4 | -32 | -2 |
| Cluster 4 (Temporal Lobe) | 19.8 | < 0.0001 | | | | |
| Planum Temporale | | | -5.74 | 62 | -22 | 6 |
| Planum Temporale | | | -5.67 | 52 | -30 | 12 |
| Superior Temporal Gyrus, posterior division | | | -5.66 | 64 | -22 | 2 |
| Parietal Operculum Cortex | | | -5.48 | 50 | -32 | 18 |
| Superior Temporal Gyrus, anterior division | | | -5.41 | 64 | -8 | -2 |
| Superior Temporal Gyrus, posterior division | | | -5.16 | 66 | -30 | 4 |
| Cluster 5 (Sub-lobar) | 19.1 | < 0.0001 | | | | |
| Cerebral White Matter | | | -6.51 | -60 | -30 | 10 |
| Cingulate Gyrus, posterior division | | | -6.14 | -50 | -40 | 16 |
| Thalamus | | | -5.28 | -44 | -20 | 10 |
| Thalamus | | | -4.88 | -56 | -12 | 2 |
| Cerebral White Matter | | | -4.76 | -44 | -24 | 16 |
| Cerebral White Matter | | | -4.54 | -64 | -10 | 14 |
| Cluster 6 (Parietal Lobe) | 5.44 | < 0.0001 | | | | |
| Supramarginal Gyrus, posterior division | | | -5.84 | 42 | -46 | 40 |
| Supramarginal Gyrus, posterior division, | | | -5.39 | 50 | -50 | 42 |
| Angular Gyrus | | | -5.14 | 38 | -52 | 32 |
| Lateral Occipital Cortex, superior division | | | -4.48 | 40 | -64 | 34 |
| Cluster 7 (Limbic Lobe) | 3.94 | < 0.0001 | | | | |
| Cingulate Gyrus, posterior division | | | -4.27 | 6 | -30 | 38 |
| Lateral Ventrical | | | -4.08 | 14 | -38 | 16 |
| Cerebral White Matter | | | -4.04 | 2 | -22 | 20 |
| Cerebral White Matter | | | -3.98 | 18 | -42 | 12 |
| Cingulate Gyrus, anterior division | | | -3.73 | 4 | -14 | 28 |
| Cingulate Gyrus, posterior division | | | -3.66 | 0 | -28 | 30 |

Coordinates (in MNI space) and Z score maxima for cluster-based statistical contrasts (all $Z > 3.1$, $p < 0.05$). Only clusters larger than 2 ml are shown. Clusters are first named by their general position

according to the Talairach Daemon, while maxima are named according to the structure with the highest probability at that position in the Harvard-Oxford Cortical and Subcortical Structural Atlases.

*SUPPLEMENTARY TABLE 6.*

*Monetary incentive delay: task characterisation and between centre examination*

| | London (n=15) | Cambridge (n=15) | Manchester (n=13) | ANOVA (n=15,15,13) | Combined (n=43) |
|---|---|---|---|---|---|
| Amount won (£) | 9.73 ± 3.26 | 10.37 ± 3.18 | 8.12 ± 1.33 | $F_{2,40} = 2.38$, $p = 0.11$ [#] | 9.47 ± 2.88 |
| Accuracy: reward (%) | 66 ± 12 | 68 ± 11 | 62 ± 6 | $F_{2,40} = 1.45$, $p = 0.25$ [#] | 66 ± 10 |
| Accuracy: neutral (%) | 63 ± 11 | 67 ± 11 | 58 ± 7 | $F_{2,40} = 2.90$, $p = 0.07$ [#] | 63 ± 11 |
| Accuracy: loss (%) | 64 ± 20 | 70 ± 21 | 50 ± 8 | *Welch's* $F_{2,23.3} = 8.00$, $p = 0.002$ [‡] | 62 ± 19 |
| Hit response time: reward (ms) | 211 ± 21 | 205 ± 25 | 224 ± 14 | $F_{2,40} = 2.97$, $p = 0.06$ | 213 ± 22 |
| Hit Response time: neutral (ms) | 215 ± 22 | 209 ± 24 | 226 ± 9 | $F_{2,40} = 2.49$, $p = 0.10$ | 216 ± 21 |
| Hit Response time: loss (ms) | 207 ± 19 | 198 ± 22 | 219 ± 8 | $F_{2,40} = 4.45$, $p = 0.018$ [‡] | 208 ± 19 |
| Striatal ROI: win anticipation > neutral anticipation (% signal) [a] | 0.28 ± 0.28 | 0.30 ± 0.27 | 0.28 ± 0.30 | $F_{2,40} = 0.02$, $p = 0.98$ | 0.28 ± 0.27 |
| Striatal ROI: win anticipation > neutral anticipation (Z score) [a] | 1.91 ± 2.05 | 2.35 ± 2.13 | 1.97 ± 2.04 | $F_{2,40} = 0.19$, $p = 0.83$ | 2.08 ± 2.04 |
| Motion (mm/s) | 0.12 ± 0.21 | 0.07 ± 0.01 | 0.07 ± 0.02 | $F_{2,40} = 0.90$, $p = 0.41$ [†] | 0.09 ± 0.13 |

[†]Normality assumptions of at least one subgroup violated (Shapiro-Wilk test), but no significant differences between groups found using a non-parametric test (Kruskal-Wallis).
[‡]Normality assumptions of at least one subgroup violated (Shapiro-Wilk test), significant differences between groups also found using a non-parametric test (Kruskal-Wallis).
[#]Normality assumptions of at least one subgroup violated (Shapiro-Wilk test), significant differences between groups also found using a non-parametric test (Kruskal-Wallis), but are not significant after a Bonferroni correction for number of independent tests performed on this task (~ 7).
[a]These values represent the means of the raw numbers extracted on an individual basis. Higher values are reported in the main text after the full mixed effects model has been carried out, and accounting for centre, age, and sex.

*SUPPLEMENTARY TABLE 7.*

| *Go/no-go: task characterisation and between centre examination* | | | | | |
|---|---|---|---|---|---|
| | London (n=15) | Cambridge (n=15) | Manchester (n=13) | ANOVA (n=15,15,13) | Combined (n=43) |
| Accuracy: go (%) | 96 ± 6 | 94 ± 19 | 97 ± 5 | $F_{2,40} = 0.53$, $p = 0.77$ [†] | 96 ± 12 |
| Accuracy: no-go (%) | 65 ± 17 | 66 ± 16 | 71 ± 15 | $F_{2,40} = 0.47$, $p = 0.63$ | 67 ± 16 |
| Go response time (ms) | 339 ± 75 | 313 ± 65 | 326 ± 57 | $F_{2,40} = 0.55$, $p = 0.58$ | 326 ± 66 |
| No-go response time (ms) | 295 ± 79 | 278 ± 85 | 274 ± 48 | $F_{2,40} = 0.33$, $p = 0.72$ [†] | 283 ± 72 |
| Striatal ROI: no-go > go (a.u.) [a] | 0.22 ± 0.12 | 0.17 ± 0.14 | 0.26 ± 0.16 | $F_{2,40} = 1.33$, $p = 0.28$ | 0.21 ± 0.14 |
| Striatal ROI: no-go > go (Z score) [a] | 1.46 ± 0.79 | 1.30 ± 0.99 | 1.41 ± 0.74 | $F_{2,40} = 0.15$, $p = 0.87$ | 1.39 ± 0.83 |
| Motion (mm/s) | 0.06 ± 0.03 | 0.06 ± 0.02 | 0.05 ± 0.02 | $F_{2,40} = 1.05$, $p = 0.36$ [†] | 0.06 ± 0.02 |

[†]Normality assumptions of at least one subgroup violated (Shapiro-Wilk test), but no significant differences between groups found using a non-parametric test (Kruskal-Wallis).
[a]These values represent the means of the raw numbers extracted on an individual basis. Higher values are reported in the main text after the full mixed effects model has been carried out, and accounting for centre, age, and sex.

“No-go response time” is that of unsuccessful no-go. a.u. = arbitrary units.

*SUPPLEMENTARY TABLE 8.*

| *Evocative images: task characterisation and between centre examination* | | | | | |
|---|---|---|---|---|---|
| | London (n=15) | Cambridge (n=15) | Manchester (n=13) | ANOVA (n=15,15,13) | Combined (n=43) |
| Neutral response time (ms) | 750 ± 242 | 587 ± 172 | 680 ± 276 | $F_{2,40} = 1.88$, $p = 0.17$ † | 672 ± 236 |
| Aversive response time (ms) | 778 ± 255 | 596 ± 157 | 696 ± 289 | $F_{2,40} = 2.20$, $p = 0.12$ † | 689 ± 244 |
| Difference in response time (aversive -neutral) (ms) | 24 ± 119 | 9 ± 67 | 15 ± 82 | *Welch's* $F_{2,25.2} = 0.14$, $p = 0.87$ | 17 ± 90 |
| Amygdala ROI: aversive > neutral (% signal) [a] | 0.31 ± 0.23 | 0.29 ± 0.26 | 0.02 ± 0.32 | $F_{2,40} = 5.06$, $p = 0.01$ ‡ | 0.21 ± 0.29 |
| Amygdala ROI: aversive > neutral (Z score) [a] | 1.54 ± 1.09 | 1.51 ± 1.13 | 0.26 ± 1.08 | $F_{2,40} = 5.98$, $p = 0.005$ | 1.15 ± 1.23 |
| Motion (mm/s) | 0.09 ± 0.03 | 0.07 ± 0.03 | 0.08 ± 0.05 | $F_{2,40} = 0.69$, $p = 0.51$ † | 0.08 ± 0.04 |

†Normality assumptions of at least one subgroup violated (Shapiro-Wilk test), but no significant differences between groups found using a non-parametric test (Kruskal-Wallis).
‡Normality assumptions of at least one subgroup violated (Shapiro-Wilk test), significant differences between groups also found using a non-parametric test (Kruskal-Wallis).
[a]These values represent the means of the raw numbers extracted on an individual basis. Higher values are reported in the main text after the full mixed effects model has been carried out, and accounting for centre, age, and sex.
